# Implementation of an Interleukin-2 National Registry: an opportunity to improve cancer outcomes

**DOI:** 10.1186/2051-1426-2-20

**Published:** 2014-06-18

**Authors:** Michael K Wong, Howard L Kaufman, Gregory A Daniels, David F McDermott, Sandra Aung, James N Lowder, Michael A Morse

**Affiliations:** 1 Department of Medicine, Suite 3455, University of Southern California, 1441 Eastlake Avenue, Los Angeles, CA 90033, USA; 2 Department of Surgery, Room 2007, Rutgers Cancer Center Institute of New Jersey, 195 Little Albany Street, New Brunswick, NJ 08901, USA; 3 Moores Cancer Center, University of California San Diego, 3855 Health Sciences Drive, La Jolla, CA 92093, USA; 4 Beth Israel Hospital Deaconess Medical Center, Masco Building, 375 Longwood Avenue, Boston, MA 02215, USA; 5 Prometheus Laboratories Inc., 9410 Carroll Park Drive, San Diego, CA 92121, USA; 6 Duke University Medical Center, Mudd Building, Rm 437, 10 Bryan Searle Drive, Durham, NC 27710, USA

**Keywords:** Interleukin-2, Immunotherapy, Cancer, Cure, Metastatic, Melanoma, Registry, Renal cell carcinoma

## Abstract

Cancer registries have proven valuable with respect to validating therapeutic safety and drug efficacy, uncovering real-world implementation practices, and their evolution over time. Modern cancer therapeutics are approved as single agents oftentimes compared to the least active approved standard agent in randomized trials. However, the burgeoning diversity and number of drugs introduces a complexity that quickly outstrips the knowledge provided by these pivotal trials. This gap in information is particularly relevant when survival is the primary therapeutic endpoint. In addition, the inherent complexity of the immune response will make registries a particularly important tool in expeditiously understanding solid tumor immunotherapy and patient outcomes.

## Background

A registry is a structured collection of observational data, records, and/or laboratory specimens that can be collected retrospectively, prospectively, or both. The specific goals of each registry vary from storing clinically qualified specimens to studying safety, patient-reported outcomes, or cost effectiveness. Registries may study the use of a particular therapy across diseases or a specific disease independent of a therapy. Unlike classic therapeutic studies, instructions regarding treatment administration are not fixed in the registry protocol and their variation is a legitimate study target. Registries may have liberal inclusion criteria but often share attributes of a formal clinical trial such as IRB approval, patient consent and site compensation for data entry. A broad cross section of participating centers spanning academic and community sites is typically included in registries.

Registries fundamentally differ from disease databases. Projects such as National Cancer Database (NCDB) and Surveillance, Epidemiology, and End Results (SEER) program are large disease-centric databases that passively collect patient demographics, death rates, and therapy history [1,2]. A therapy-specific registry collects additional data aiming to define and change treatment paradigms and positively impact patient morbidity, quality of life, and survival. The longitudinal follow-up of a broad patient population treated in a real world setting exposes rare or late toxicities, serves as source material to help generate hypotheses about optimal sequencing or combination of therapies, permits assessment of subject outcomes, and allows for prospective testing and validation of novel ideas. Registries also have an advantage over randomized clinical trials since they continually update and permit real-time analysis to address questions as they arise. Thus, registries occupy a unique but critical position in oncology care.

Therapy-specific registries have a track record of transforming medical practice. The Center for International Bone Marrow Transplant Registry (CIBMTR) is the prototype for how a registry globally influenced the evolution of a therapy and positively impacted patient outcomes [3,4]. Specific examples of CIBMTR’s influence include; the refinement of preparative regimens, the supportive measures necessary to maintain the patient during neutropenia, the understanding of the MHC system to match donor/recipient, refinement of immunosuppression to manage graft versus host disease, the value of transplanting in remission and the use of unrelated donors. Outcomes continued to improve and transplant use increased despite tectonic shifts in indications for BMT, and the advent of oral targeted therapy (i.e. imatinib, dasatinib) for diseases previously only treatable with transplant.

A disease-specific registry for mRCC, The International Metastatic Renal-Cell Cancer Database Consortium (IMRDC) has published a number of reports since 2009 regarding prognostic models, sequencing of agents, and conditional survival for treatment with targeted therapy agents [5-7]. A unique feature of mRCC therapy has been the rapid introduction of multiple agents of similar class. The IMRDC registry observed that no meaningful differences in survival are associated with any particular sequence of VEGF and mTOR targeted therapies [6], yet at least 4 randomized clinical trials have been performed demonstrating this point [8-11].

Clearly there are limitations to registries. Retrospective data can be biased in multiple ways including: nonconsecutive patient selection, lower performance status patients may opt out of entering the study, heterogeneous patient populations, differences in practice patterns, variations in documentation and data abstraction, and absence of adjustments for subsequent treatments which might affect overall survival. All these potential pitfalls were considered in the construction and conduct of the interleukin-2 (IL-2) registry discussed below.

### Interleukin-2 immunotherapy is a high impact registry opportunity

Cancer immunotherapy, such as HD IL-2, consistently delivers durable long term, therapy-free responses. The combination and concurrent use of emergent new therapies may enhance but also potentially threaten this curative potential. There is a critical knowledge gap about how best to sequence new drugs or to incorporate them into existing therapy choices to maximize long term patient outcomes. Immune checkpoint inhibitors add another potentially curative therapy whose interaction with IL-2 and other therapies is complex. Long-term survival and toxicity data are already available for IL-2, thus providing a reference point against which to interpret the impact of new therapies in sequence or combination. Furthermore, the availability of HD IL-2 therapy is limited by the highly technical nature of its administration due to its toxicity [12,13]. A registry is an important resource in helping establish best practices for IL-2 therapy as new agents are improved for the treatment of melanoma and renal cell carcinoma.

With these aims firmly in mind, the **Pr**oleukin® **O**bservational Registry to Evaluate the Treatment Patterns and **Cl**inic**a**l Response in **M**alignancy (PROCLAIM^SM^)(http://www.proclaimregistry.com) was created in 2011, with both retrospective and prospective cohorts. PROCLAIM establishes a clinical database on patients diagnosed with advanced kidney cancer (mRCC) and melanoma (mM) who are treated with HD IL-2 [14]. Data points include: patient demographics, peri-treatment adverse events and management, other therapies, prior, during and after the IL-2. Outcomes, including long term survival will be collected. A major goal of PROCLAIM is to serve as a resource for interested investigators to generate new ideas and to answer high priority scientific and clinical questions. Presently, there are over 35 sites participating in the PROCLAIM Registry and over 900 patients enrolled. PROCLAIM has generated a number of national meeting presentations [15,16] and recently released its second Annual Report [17]. Analyses with regards to toxicity, response rates, outcomes and optimal drug sequence are in progress. These will have significant impact on patient selection, toxicity management and most importantly, the ‘cure’ rate for these cancers.

## Conclusion

Efficiently refining the role of marketed cancer therapeutics demands that investigators generate hypotheses with the greatest potential for decisive answers and ‘real-life’ clinical utility. We believe that this can only be achieved by registries, including PROCLAIM, real-time, real-use sources of structured information which supply the raw data from which testable hypotheses arise. PROCLAIM serves this critical role by providing the data from which any interested investigator could generate new questions about high dose IL-2 therapy, including those that will establish modern benchmarks for outcomes and create best practices. Structured to support a productive collaboration between industry, academia, and community practice, PROCLAIM will serve as a model for other registries in rapidly evolving areas of oncology therapeutics where there is a pressing need to integrate more established drugs with new therapeutics.

## Competing interests

The authors MW, HK, DM, MM, and GD serve as consultants (and speaker and advisory board members) for Prometheus Laboratories Inc., which markets interleukin-2 (Proleukin), the product related to the article content.

## Authors’ contributions

MW and MM both wrote and revised the manuscript, with editorial assistance from HK, MD, GD, JL, and SA. All authors read and approved the final manuscript.
